# American Medical Association and Convention of Medical Teachers

**Published:** 1860-07

**Authors:** 


**American Medical Association and Convention of Medical Teachers.**

It is, perhaps, to be regretted that, hitherto, every attempt to procure a *general* representation of the Medical Colleges, Associations and Societies, scattered throughout the Northern, Southern, Eastern and Western sections of our great Republic, has been signally unsuccessful. Nor does the recent assemblage at New Haven, wearing the imposing title of the "American Medical Association and Convention of Medical Colleges," form any exception, in this particular, to the history of its previous sessions. The Atlanta Medical College, however, as has been her custom, was represented there. But only twenty others, out of the forty or more respectable Institutions of this grade within the limits of the United States, sent delegates to the Convention of Colleges, and only about *thirty* members, out of the entire assemblage in the "Association," of, perhaps, five hundred, were from the South; while not one of the oldest and most popular Colleges in the land, had a representation in the Convention of Teachers, or, so far as we know, in the "Association."

This is not as it should be, in order to maintain the deserved prestige and influence of the Association, and secure harmony of action upon the great professional questions, which have for several years past occupied so much of its time and attention. From the defective and partial representation above referred to, it is almost impossible clearly to ascertain what is the popular animus of the profession throughout this broad land, and to settle, without assumption and arrogance, all the perplexing points at issue.

That honest differences of opinion might, under any circumstances, characterize the deliberations of all such bodies, however ably represented, is readily conceded, but then a numerical preponderance in the vote, would carry with it the sanction, at least, of conventional law.

Whatever, then, may *now*, or in *future*, be adopted and sustained by the great body of the Medical profession, and especially in the South, where she records her birth, and whence she receives her liberal patronage, will meet with the hearty co-operation of the Atlanta Medical College, and her patrons and friends may rest assured that she will never stoop to ignoble methods to secure her prosperity, not even take one step backward from her hitherto settled purpose and unswerving course to maintain the dignity, elevate the standard, and enlarge the circle of scientific and practical medicine.

